# Human histamine H_2_ receptors can initiate cardiac arrhythmias in a transgenic mouse

**DOI:** 10.1007/s00210-021-02098-y

**Published:** 2021-06-24

**Authors:** U. Gergs, J. Weisgut, K. Griethe, N. Mißlinger, U. Kirchhefer, Joachim Neumann

**Affiliations:** 1 Institute for Pharmacology and Toxicology, Medical Faculty, Martin Luther University Halle-Wittenberg, Halle (Saale), Germany; 2 Institute for Pharmacology and Toxicology, University Hospital Münster, Westfälische Wilhelms University, Münster, Germany; 3 Institut Für Pharmakologie Und Toxikologie, Medizinische Fakultät, Martin-Luther-Universität Halle-Wittenberg, Magdeburger Str. 4, 06112 Halle (Saale), Germany

**Keywords:** Cardiac arrhythmias, Histamine, Histamine receptor, Transgenic mouse

## Abstract

Histamine is known to lead to arrhythmias in the human heart. A mouse model to mimic these effects has hitherto not been available but might be useful to study the mechanism(s) of H_2_-histamine receptor-induced arrhythmias and may support the search for new antiarrhythmic drugs. In order to establish such a model in mice, we studied here the incidence of cardiac arrhythmias under basal and under stimulated conditions in atrial and ventricular preparations from mice that overexpressed the human H_2_-histamine receptors in a cardiac-specific way (H_2_-TG) in comparison with their wild-type (WT) littermate controls. We had shown before that histamine exerted concentration and time-dependent positive inotropic and positive chronotropic effects only in cardiac preparations from H_2_-TG and not from WT. We noted under basal conditions (no drug addition) that right atrial preparations from H_2_-TG exhibited more spontaneous arrhythmias than right atrial preparations from WT. These arrhythmias in H_2_-TG could be blocked by the H_2_-histamine receptor antagonist cimetidine. In a similar fashion, histamine and dimaprit (an agonist at H_2_ and not H_1_-histamine receptors) more often induced arrhythmias in right atrial preparations from H_2_-TG than from WT. To understand better the signal transduction mechanism(s) involved in these arrhythmias, we studied partially depolarized left atrial preparations. In these preparations, a positive inotropic effect of histamine was still present in the additional presence of 44 mM potassium ions (used to block sodium channels) in H_2_-TG but not WT and this positive inotropic effect could be blocked by cimetidine and this is consistent with the involvement of calcium ion channels in the contractile and thus might mediate also the arrhythmogenic effects of histamine in H_2_-TG. However, compounds reported to release histamine from cells and thereby leading to arrhythmias in humans, namely morphine, ketamine, and fentanyl, failed to induce a more pronounced positive inotropic effect in atrial preparations from H_2_-TG compared to WT, arguing against an involvement of histamine release in their proarrhythmic side effects in patients. Measuring left ventricular contractility in isolated retrogradely perfused hearts (Langendorff mode), we detected under basal conditions (no drug application) more spontaneous arrhythmias in hearts from H_2_-TG than from WT. In summary, we noted that overexpression of human H_2_-histamine receptors in a novel transgenic animal model can lead to arrhythmias. We suggest that this model might be useful to understand the mechanism(s) of histamine-induced cardiac arrhythmias in humans better in a molecular way and may be of value to screen novel antiarrhythmic drugs.

**Supplementary Information:**

The online version contains supplementary material available at 10.1007/s00210-021-02098-y.

## Introduction

**Fig. 1:**
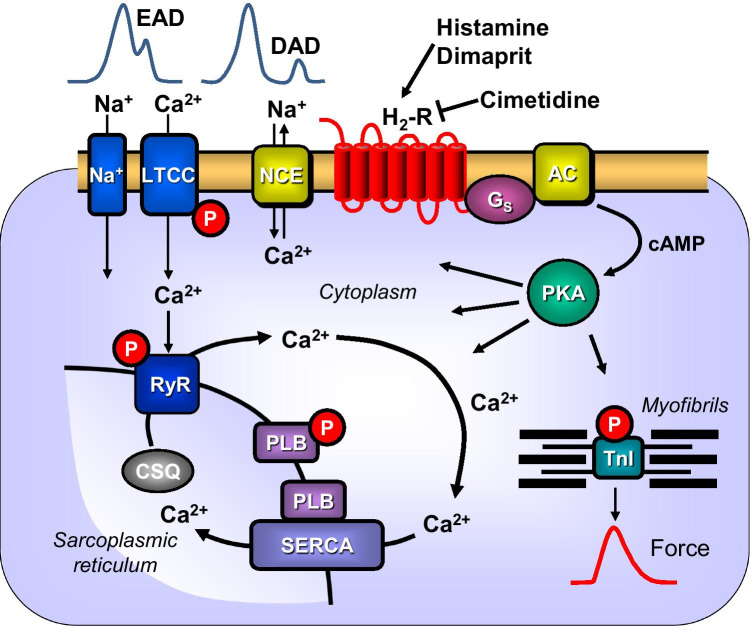
Scheme of cardiomyocytes. Histamine or its derivative dimaprit can activate H_2_-histamine receptors via stimulatory GTP binding proteins (Gs), an adenylate cyclase (AC) which leads to the production of 3′, 5′-cyclic adenosine mono phosphate (cAMP). Thereafter, cAMP-dependent protein kinase (PKA) can phosphorylate and activate the ryanodine receptor 2 (RYR), the L-type Ca^2+^ channel (LTCC), phospholamban (PLB), and the inhibitory subunit of troponin (TnI). Ca^2+^ is pumped from the cytosol into the sarcoplasmic reticulum via the sarcoplasmic reticulum Ca^2+^ ATPase (SERCA2a). SERCA2a activity is increased if phospholamban is phosphorylated by PKA. Ca^2+^ in the sarcoplasmic reticulum is bound to calsequestrin (CSQ). An increase in force is thought to result from an increase in cytosolic Ca^2+^. This Ca^2+^ can be extruded from the cell via the electrogenic sodium/calcium exchanger (NCE): This leads to muscle depolarization which can result in early (EAD) and late afterdepolarizations (DAD). Cimetidine antagonizes the H_2_-histamine receptor

Histamine is a naturally occurring monoamine studied for decades in many biological systems (Parsons and Ganellin 2006; Haas et al. 2008). Histamine was first synthesized by chemists in Freiburg without knowing its physiological function (Windaus and Vogt, 1907). Mast cells, present also in human hearts, contain large concentrations of histamine, but minor levels of histamine are found in most cells investigated (Jutel et al. 2009). More recently, we presented evidence that histamine can be formed and degraded in cardiomyocytes (Gergs et al. 2016; Neumann et al. 2021b). Histamine can be taken up into the body via the gastrointestinal tract to some extent, but can also be formed from histidine by the enzyme histidine decarboxylase which is present in many cells of the human body. Currently, the effects of histamine are thought to be mediated by four different receptors known as H_1_-, H_2_-, H_3_-, and H_4_ -histamine receptors (for review, see Seifert et al. 2013; Panula et al. 2015). In isolated muscle preparations of the human heart, a positive inotropic effect to histamine was observed that led to an increase in cAMP (Fig. 1), an activation of cAMP-depending protein kinase (human right atrial preparations: Sanders et al. 1996), opening of L-type Ca^2+^ channels (Eckel et al. 1982) and this positive inotropic is thought to be H_2_-histamine receptor mediated (Wolff and Levi 1986, see scheme in Fig. 1).

Arrhythmogenic effects of histamine have been published within 3 years after the first reports on cardiovascular effects of histamine. Asystole after giving histamine in isolated perfused frog hearts and third-degree atrioventricular heart block in isolated rabbit hearts were noted (Einis 1913). In the same year, a German gynecologist studied histamine in postpartal women to contract the uterus and in order to stop bleeding. He reported (at a scientific meeting at our university in Halle, Germany) that in some of the histamine-treated women, palpitations occurred, indicating that he induced cardiac arrhythmias by injecting histamine (Jäger 1913a, b). Using body surface electrocardiograms, Schenk likewise reported on ventricular extrasystoles in the heart of patients after injections of histamine (Schenk 1921).

Decades later using more advanced methods, infusions of histamine into the arteria brachialis of human volunteers led to multiple forms of atrioventricular blocks and ventricular idiopathic rhythms and these rhythm disturbances turned out to be reversible upon termination of the infusion of histamine (Vigorito et al. 1983).

Cardiac arrhythmias have also been connected with histamine when studying ischemia and reperfusion. Reperfusion can lead to elevated histamine levels in the heart and this histamine has been suggested to induce arrhythmias in laboratory animals and extension of these animal findings might be relevant in humans (review: Wolff and Levi 1986). To cite more recent work, in isolated perfused wild-type mouse hearts, reperfusion in the Langendorff mode led to arrhythmias (He et al. 2012). These arrhythmias (including ventricular arrhythmias) were not blocked by pretreatment of wild-type hearts with famotidine (an H_2_-histamine receptor antagonist) alone or atenolol (a β-adrenoceptor antagonist) alone but only by their combination (He et al. 2012). On the other hand, addition of isoprenaline (a β-adrenoceptor agonist) or histamine to the isolated mouse hearts led in nearly all experiments to cardiac arrhythmias (He et al. 2012): When these authors used isolated perfused hearts from HDC-KO animals (from mice with global histidine decarboxylase knockout, where no histamine could be formed because HDC is the pace-making enzyme for histamine formation in vivo), they detected far less, if any, arrhythmias in these isolated perfused mouse hearts after reperfusion (He et al. 2012). However, these results are in contrast to our own reports: We never detected inotropic or chronotropic effects of histamine in living mice, isolated mouse heart preparations but only in H2-transgenic mice (Gergs et al. 2019, 2020). In patients, higher plasma levels of histamine correlated with the appearance of atrial fibrillation (Layritz et al. 2014). Red wine contains higher amounts of histamine than white wine and indeed there are studies connecting red wine intake and an increased incidence of arrhythmias in human populations (Liang et al. 2012). Hence, histamine-induced arrhythmias might present an underinvestigated source of cardiac disease.

Fittingly, there are case reports about patients with allergic anaphylaxis after consumption of fish or kiwi fruits (both of which can contain high levels of histamine and/or histidine led to cardiac arrhythmias) (Rojas-Perez-Ezquerra et al. 2017). In children and adults with mastocytosis (a rare disease with a genetically caused increase in mast cells and thus histamine levels in the human body), a high incidence of arrhythmias was reported (Rohr et al. 2005, Shaffer et al. 2006). In addition, morphine and morphine-like compounds like fentanyl or other strong analgesics like ketamine can release histamine and this might explain in part why they can induce cardiac arrhythmias in some patients.

Histamine could induce in isolated right atrial human tissue arrhythmias which were accompanied by and possibly caused by delayed afterdepolarizations (Levi et al. 1981, see scheme in Fig. 1). These arrhythmias could be blocked by famotidine (but not by mepyramine, a H_1_-histamine receptor antagonist) and were thereby regarded as H_2_-histamine receptor mediated (Sanders et al. 1996). Hitherto, no animal model is available to study the involvement of human H_2_-histamine receptors. For instance, both H_1_ and H_2_-histamine receptors are functionally active in guinea pig left atrium and guinea pig ventricle. Likewise, in rabbits, H_1_- and H_2_-histamine receptors are functionally active in the left atrium, right atrium, and ventricle (Hattori et al. 1994). Solely in guinea pig right atrium, an H_2_-histamine receptor is mainly active (Reinhardt et al. 1977) but has a different primary sequence than the human H_2_-histamine receptor. In rat, dog, and cat, inotropic effects of histamine were found to be indirect (Flacke et al. 1967; Dai 1976; Bartlet 1963; Wellner-Kienitz et al. 2003): that is, via release of endogenous catecholamines (Laher und McNeill 1980a, 1980b). Thus, histamine can cause arrhythmias via human H_2_-histamine receptors, but in the present work, we wanted to know whether activation of human H_2_-histamine receptors in a transgenic mouse model might also induce arrhythmias and whether these arrhythmias are blocked by H_2_-histamine receptor antagonists, whether they involve sarcolemmal Ca^2+^ channels, whether these arrhythmias occur only in atrium or only in the ventricle, and whether in this model analgetic drugs like ketamine, fentanyl, and morphine, reported to be arrhythmogenic in some patients, might activate H_2_-histamine receptors, a mechanism that might explain their proarrhythmic side effects. Initial data of the present work were published in abstract form (Weisgut et al. 2015; Neumann et al. 2015; Griethe et al. 2016; Gergs et al. 2017).

## Methods

### Transgenic mice

The investigation conforms to the *Guide for the Care and Use of Laboratory Animals* published by the National Research Council (2011). Animals were maintained and handled according to approved protocols of the animal welfare committees of the Martin Luther University of Halle-Wittenberg, Germany. The generation and initial characterization of mice with cardiac-specific overexpression have been reported from our group (Gergs et al. 2019, 2020; Neumann et al. 2021b). We used an α-myosin heavy chain promoter to overexpress the human H_2_-histamine receptor in the mouse heart and tested the offspring for transgenes using the polymerase chain reaction (Gergs et al. 2019, 2020; Neumann et al. 2021b).

### Contractile function

Mice were anesthetized by i.p. injection of pentobarbital sodium (50 mg kg^−1^) and hearts were excised. Right and left atria were dissected from isolated H_2_-histamine receptor transgenic and wild-type mouse hearts and mounted in an organ bath. Left atrial preparations were continuously electrically stimulated (field stimulation) at 1 Hz, with a voltage of 10–15% above threshold and 5 ms duration. Right atrial preparations were allowed to contract spontaneously. The bathing solution contained (in mM) NaCI 119.8, KCI 5.4, CaCl_2_ 1.8, MgCl_2_ 1.05, NaH_2_P0_4_ 0.42, NaHCO_3_ 22.6, Na_2_EDTA 0.05, ascorbic acid 0.28, and glucose 5.0, continuously gassed with 95% O_2_ and 5% CO_2_ and maintained at 35 °C resulting in a pH of 7.4. In some experiments, we increased the potassium chloride concentration to 44 mM without taking into consideration the higher osmotic pressure under these conditions, because we studied H_2_-TG and WT preparation in direct comparison and hence increased osmotic pressure cannot explain the difference we observed between H_2_-TG and WT preparations.

Signals detected via an isometric force transducer were amplified and continuously recorded using a PowerLab system (ADInstruments, Oxford, UK) as published (Gergs et al. 2019, 2020).

### Langendorff-perfused hearts

Mouse heart preparations were utilized as described previously (Gergs et al. 2019, 2020; Neumann et al. 2021b). Basically, we were using the classical Langendorff method for isolated mammalian heart perfusion (Langendorff 1895). Mice were anesthetized intraperitoneally with pentobarbital sodium (50 mg kg^−1^) and treated with 1.5 units of heparin. The hearts were removed from the opened chest, immediately attached by the aorta to a 20-gauge cannula, and perfused retrogradely under constant flow of 2 ml min^−1^ with oxygenized buffer solution (37 °C) containing (in mM) NaCI 119.8, KCI 5.4, CaCl_2_ 1.8, MgCl_2_ 1.05, NaH_2_P0_4_ 0.42, NaHCO_3_ 22.6, Na_2_EDTA 0.05, ascorbic acid 0.28, and glucose 5.0 in an isolated heart system manufactured by our in-house technical shop. The heart preparations were allowed to equilibrate for 30 min before measurements. Developed force was taken from the apex cordis and fed via a silk thread into an isometric force transducer connected to a bridge amplifier. The developed force and the first derivative of force with regard to time (+ dF/dt and − dF/dt) were processed using a PowerLab system (ADInstruments, Oxford, UK).

### Data analysis

Data shown are means ± SEM. Statistical significance was estimated by paired or unpaired t-tests, analysis of variance followed by Bonferroni´s t-test or using the χ^2^ test as appropriate. A p-value < 0.05 was considered as significant.

### Drugs and materials

All chemicals were of analytical grade. Demineralized water was used throughout the experiments. Stock solutions were freshly prepared daily.

## Results

We have shown previously that histamine is able to increase the beating rate (and force of contraction) in a time and concentration-dependent manner in atrial and ventricular preparations from H_2_-TG but not WT (Gergs et al. 2019, 2020, Neumann et al. 2021a). Even if we did not add histamine to the organ bath or the perfused heart, the beating rate in H_2_-TG was higher than in WT (Gergs et al. 2019, 2020, Neumann et al. 2021a).

**Fig. 2:**
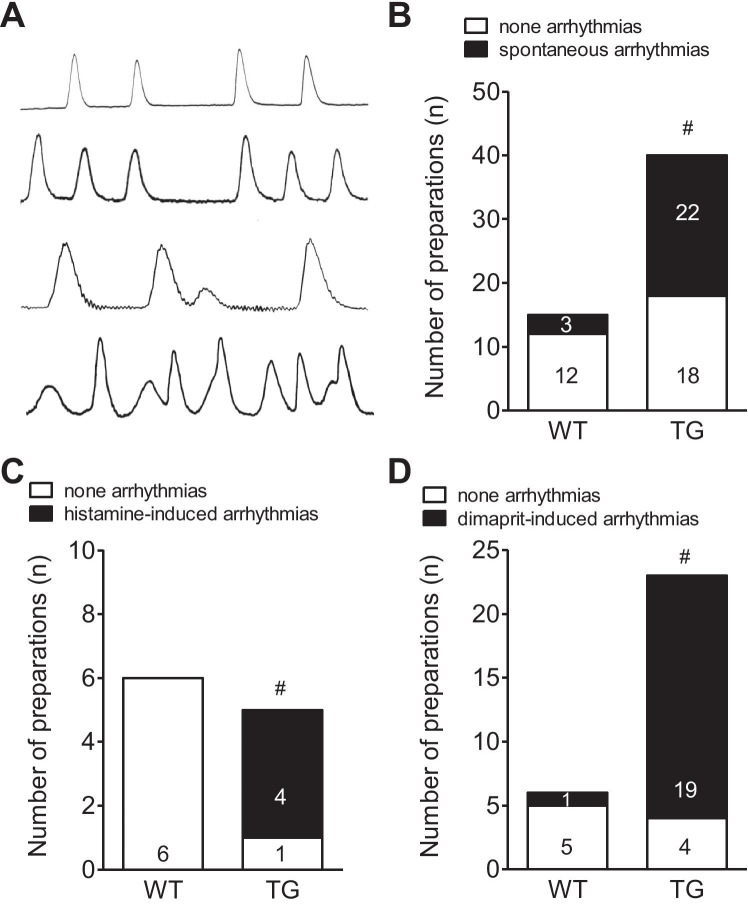
**A** Representative examples of dimaprit (1 µM)-induced arrhythmias in isolated spontaneously beating right atrial preparations. Uppermost lane was from WT mice and the other lanes from TG mice. **B**-**D** Ordinates indicate the number of right atrial preparations from WT mice and H_2_-TG mice (TG). Open space in bars indicates the number of right atrial preparations without arrhythmias and closed parts of the bars indicate the number of samples with arrhythmias. Spontaneous arrhythmias are plotted in **B**, histamine-induced arrhythmias are depicted in **C**, and dimaprit-induced arrhythmias are seen in **D**. ^#^p < 0.05 vs. WT

We never noted atrial arrhythmias in isolated electrically driven left atrial preparations from H_2_-TG or WT (1 Hz, data not shown). However, in right atrial preparations, we noted arrhythmias. Typical original tracings are seen in Fig. 2A. Under basal conditions (no drug addition), very few arrhythmias were noted in right atrial preparations in WT, while arrhythmias were noted in H_2_-TG (Fig. 2B). Spontaneous arrhythmias in right atrial preparations of TG were cimetidine (10 µM) sensitive (3/3, p < 0.05 vs. WT). In those right atrial preparations, that did not exhibit spontaneous arrhythmias, 1 µM histamine (as reported before: Gergs et al. 2019, 2020; Neumann et al. 2021a, b). No arrhythmias were noted in WT, whereas a significant increase in the incidence of arrhythmias was detected in H_2_-TG (Fig. 2C). In another set of experiments, dimaprit (1 µM as in Gergs et al. 2019) induced less arrhythmias in WT than in H_2_-TG (Fig. 2D). The dimaprit-induced arrhythmias in isolated right atrial preparations of TG could be attenuated by the H_2_-histamine receptor antagonist cimetidine (10 µM, 4/4, p < 0.05 vs. WT).

**Fig. 3:**
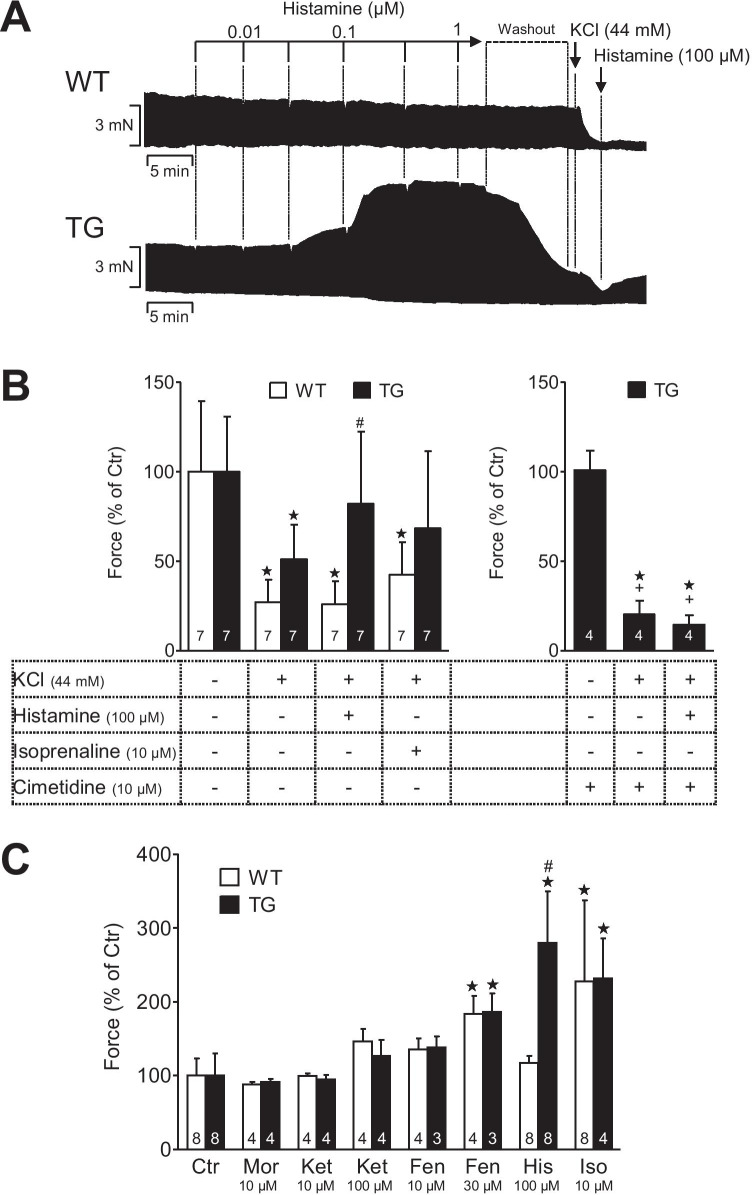
**A** Typical original tracings of electrically stimulated (1 Hz) left atrial preparations are shown. One can see that histamine increases force of contraction only in H_2_-TG (TG) and that high potassium (44 mM) reduces force of contraction in both wild type (WT) and H_2_-TG. Additionally applied 100 µM histamine could increase force of contraction in H2-TG but not in WT. **B**, **C** Ordinates indicate force of contraction of isolated left atrial preparations in % of pre-drug values. Potassium ions (44 mM KCl bath concentration) were added to partially depolarize left atrial preparations stimulated at 1 Hz of WT and H_2_-TG (TG) and thus reduced force of contraction. Histamine induced a positive inotropic effect in H_2_-TG but not in WT, whereas isoprenaline (Iso) increased force of contraction in both WT and H_2_-TG. Histamine induced a positive inotropic effect in H_2_-TG which was antagonized by cimetidine. **C** Fentanyl (Fen), ketamine (Ket), morphine (Mor) or histamine (His), or isoprenaline (Iso) were added with the final bath concentrations indicated in µM under the bars. Solvent control is also indicated (Ctr). Numbers in brackets indicate number of experiments. *p ˂ 0.05 vs. Ctr; #p ˂ 0.05 vs. WT; + *p* < 0.05 vs TG without cimetidine

To get more insight into the possible mechanism(s) of these arrhythmias, the concentration of potassium ions was increased in the organ bath to 44 mM: a procedure intended to partially depolarize the samples. A 44-mM potassium ion concentration reduced force of contraction in left atrial preparations (stimulated at 1 Hz) of both WT and H_2_-TG substantially (original tracing and bar diagram in Fig. 3). Additionally applied histamine increased force of contraction only in H_2_-TG and not in WT (original tracing and bar diagram in Fig. 3). Isoprenaline in contrast increased force of contraction in both WT and H_2_-TG (Fig. 3B), suggesting an involvement of the L-type Ca^2+^ channel (Fig. 1) in these atrial preparations in the signal transduction pathway of histamine. The histamine-induced increase in force in potassium-treated samples was blocked by cimetidine (Fig. 3B), suggesting that the inotropic effect of histamine in depolarized atrial preparations was due to H_2_-histamine receptor activation. Morphine (the highest concentration tested: 10 µM) slightly, but not significantly, reduced force of contraction in left atrial preparations, but to the same extent in WT and H_2_-TG (Fig. 3C), arguing against a relevant morphine-induced histamine release from left atrial preparations in H_2_-TG. Ketamine at 10 µM did not alter force of contraction in left atrial preparations from WT or H_2_-TG. However, higher concentrations of 100 µM ketamine increased force of contraction in left atrial preparations from WT or H_2_-TG, but to a similar extent in WT than in H_2_-TG (Fig. 3C), also suggesting that ketamine did not release histamine because then the positive inotropic effect of ketamine should be larger in H_2_-TG than in WT. Moreover, in additional experiments, we noted that the positive inotropic effect of ketamine at 100 µM was blocked by 10 µM propranolol but not by 10 µM cimetidine (n = 3–4 each, data not shown), suggesting that an indirect effect was present: Ketamine might have released noradrenaline. Like ketamine, fentanyl increased concentration-dependent force of contraction in left atrial preparations but to the same extent in WT and H_2_-TG. Moreover, in additional experiments, we noted that the positive inotropic effect of fentanyl at 10 µM and 30 µM was not blocked by 10 µM cimetidine or 10 µM propranolol (n = 3–4 each, data not shown), arguing against a fentanyl-induced release of histamine which should only manifest itself in H_2_-TG and be blocked by cimetidine and also arguing against a release of noradrenaline the effect of which on β-adrenoceptors would be antagonized by propranolol. In addition, vancomycin is well known to release histamine and clinically it has been related to the “red man syndrome” (Martel et al. 2021). Hence, we also studied vancomycin. However, at 10 µM and 30 µM, vancomycin failed under our conditions to increase force of contraction in left atrial preparations from H_2_-TG or WT (n = 3 each, data not shown).

**Fig. 4:**
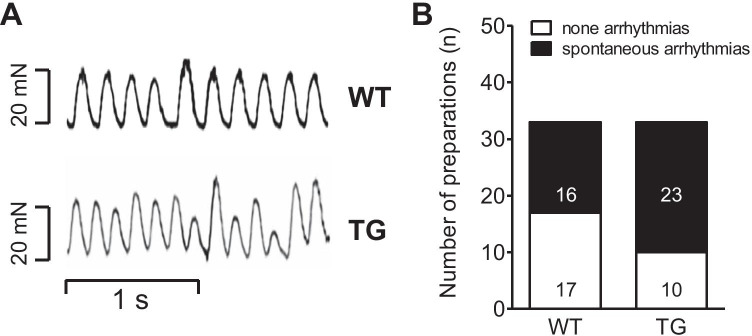
Spontaneously induced arrhythmias in isolated Langendorff hearts in WT mice and H_2_-TG mice (TG). **A** Original recordings depict perfused hearts from WT (top) and H_2_-TG (bottom). **B** Open space in bars indicates the number of right atrial preparations without arrhythmias and closed parts of the bars indicate the number of samples with arrhythmias. The incidence of arrhythmias is higher in H_2_-TG than in WT (*p* < 0.05). Horizontal line indicates time axis. Vertical line indicates scale of force of contraction in milli Newton (mN)

Finally, one can ask how ventricular rhythm is affected in H_2_-TG compared to WT under basal conditions. It turned out that under our experimental conditions (isolated buffer retrogradely perfused hearts in the Langendorff mode), more basal arrhythmias (original tracings in Fig. 4A) were noted in H_2_-TG than in WT as summarized in Fig. 4B. In the original recordings, one can detect late irregular contractions that are consistent with an involvement of late afterdepolarizations (Fig. 4A).

## Discussion

The main new finding of the current work is that overexpressed human H_2_-histamine receptors per se can lead to cardiac arrhythmias in a new mouse model (H_2_-TG). One can ask how endogenous H_1_ and H_2_ receptors are expressed in the wild-type mouse heart and in H_2_-TG. We have detected endogenous mouse H_2_ (and H_1_) receptors in the mouse heart, when one prepares RNA from total mouse heart tissue (Neumann et al. 2017). Hence, the mRNA of the mouse H_2_ receptor is certainly present in the heart. However, we have not succeeded in detection by PCR of mouse H_2_ receptors in RNA preparations from adult mouse cardiomyocytes (unpublished observations). Hence, it is clear that the RNA for the mouse H_2_ receptor exists in the total mouse heart; however, it is presently unclear whether it is present in wild-type cardiomyocytes or perhaps in other cardiac cells like endothelial cells, fibrocytes or smooth muscle cells, or even mast cells. Moreover, for the receptor to function, the H_2_ receptor has to be present on a protein level not only on the RNA level. We worked hard on this issue but could not find a commercial antibody that selectively detects the mouse or human H_2_ receptor. This is a well-known problem with G-protein-coupled receptors (Seifert et al. 2013) which we mentioned openly when we first published the generation of the H_2_-TG (Gergs et al. 2019). Finally, and to us most importantly, we cannot detect a positive inotropic or chronotropic effect in wild-type mice to histamine or dimaprit in several publications in wild-type mouse hearts (Gergs et al. 2019, 2020; Neumann et al. 2021a, b). Moreover, using a radiolabeled H_2_ receptor ligand, we could detect the H_2_ receptor in H_2_-TG by autoradiography but not by ligand binding in membrane preparations (Gergs et al. 2019) which suggests to us that the expression of endogenous H_2_ receptors in mouse hearts is low and even transgenic human H_2_ receptors are sparsely expressed on the protein level, the latter being functional.

### Experimental findings

As concerns the mechanism of the genesis of arrhythmias in the present model, a role of intracellular Ca^2+^ seems probable. This might manifest itself in early or late afterpolarizations both of which would lead to electrical and mechanical arrhythmias (Fig. 1). We have reported before that histamine elevates Ca^2+^ in ventricular cardiomyocytes from H_2_-TG but not WT (Gergs et al. 2019). New evidence produced here is that Ca^2+^ might also be involved in atrial arrhythmias. We present indirect evidence for this conclusion. We have used partially depolarized left atrial preparations (by increasing potassium ion concentration in the organ bath) and noted that histamine can still induce increases in force of contraction in these atria from H_2_-TG but not WT and that this effect is seen when one uses dimaprit (which mainly acts at H_2_-, and, to some extent, also at H_3_- and H_4_-histamine receptors but not at H_1_-histamine receptors: review: Panula et al. 2015) and that these effects are blocked by cimetidine (which only blocks H_2_ but not other histamine receptors, Panula et al. 2015). In guinea pig, tissue partial depolarization of cardiac tissue by potassium has been used before to study indirectly an action of histamine (Levi und Giotti 1967; Levi und Pappano 1978). Thus, we hypothesize that in atrial tissue, L-type calcium channel histamine via (Fig. 1) H_2_-histamine receptors leads to inflow of Ca^2+^ through the L-type calcium channel and this increases in cytosolic Ca^2+^, for instance, by being pumped outside of the cell through the sodium calcium exchanger depolarizes the cell and thus induces arrhythmia (Fig. 1).

Morphine, fentanyl, and ketamine can cause arrhythmia in humans (Hickey and Hansen 1991, Behzadi et al. 2018; Emerling et al. 2020, respectively). In agreement with our current findings, a small negative inotropic effect of morphine has been reported before (e.g., rat atrium Helgesen and Refsum 1987). It is well known that morphine can release histamine from mast cells in vitro (Ginsburg et al. 1981; Moss and Rosow 1983). Indeed, morphine has been reported to release histamine from the human heart (Levi et al. 1982). Ketamine has also been reported to release histamine from the hearts (cat: Costa-Farré et al. 2005). Ketamine has been suggested to release histamine in humans (Bylund et al. 2017). In contrast to morphine and fentanyl, ketamine is used as anesthetic drug and acts via blocking NMDA receptors and not via morphine receptors (Kohrs and Durieux. 1998). Based on the aforementioned literature, we hypothesized at the start of the present study that morphine receptor agonists might release histamine in mouse atrial preparations and this histamine was expected to increase force in the left atrium of H_2_-TG but not in WT by increasing cytosolic Ca^2+^ and thus lead to arrhythmias. However, this was not the case. Ketamine has likewise been shown to exert a slight positive inotropic effect (right atrial preparations from patients: Kunst et al. 1999; rat atrium: Endou et al. 1992). It can be asked why we chose to test these compounds in left atrial preparations of mice. Actually, we came across papers that morphine can release histamine and that this might lead to arrhythmias in patients. We wanted to test this observation in our model. If morphine really released histamine and this cardiac release of histamine can activate cardiac H_2_ receptors in humans, this would be a reasonable chain of events. Hence, it seemed a logical first step to test the contractile effects of morphine in H_2_-TG compared to WT. If the inotropic effects of morphine are lacking in WT (which do not respond to histamine) but are present in H_2_-TG (which respond to histamine) and are blocked by cimetidine (indicating that they are truly H_2_-mediated), then we would have went further and have studied their ability to induce arrhythmias in right atrial preparations of H_2_-TG and WT (for control). However, as morphine did not increase force of contraction in H_2_-TG nor in WT, the initial hypothesis could be rejected: Morphine is not able to release histamine in the mouse heart in a concentration that could increase force of contraction. Hence, we did not go further to study morphine in right atrial preparations to elicit arrhythmias.

At least in cats, fentanyl has been suggested to induce histamine release and subsequent vasodilatation and reduction of blood pressure (Kaye et al. 2006). In support of our present results on contractility of fentanyl, a small positive inotropic effect of fentanyl has also been noted before (isolated perfused rat heart: Gürkan et al. 2005). However, the inotropic effect of fentanyl was not confined to left atria from H2-TG but also seen in WT and was not blocked by cimetidine. However, the positive inotropic effects of fentanyl were also not blocked by propranolol (an unselective β-adrenoceptor antagonist) and thus release of noradrenaline cannot explain the inotropic effect of fentanyl. The mechanism of this effect waits to be elucidated.

It is possible that the level of histamine released by morphine, fentanyl, or ketamine in mouse left atrium was too low to affect force of contraction or more likely that the number of mast cells in mouse hearts is too low to release measurable amounts of histamine or that mast cells in the heart are not sensitive to morphine and ketamine and fentanyl in contrast to skin mast cells (Patella et al. 1995, Theoharides et al. 2011). However, in principle, the current H_2_-TG is able to release histamine and react with a positive inotropic effect: We have shown that using a typical histamine-releasing compound 48/80 (Meister et al. 2015).

## Clinical relevance

The present data clearly indicate that H_2_-histamine receptor overexpression per se is arrhythmogenic in the atrium as well as the ventricle. It is conceivable (but apparently has not yet been investigated) that an increase in the density of H_2_-histamine receptors might occur and might underlie arrhythmias in some patients. In these patients, applying an H_2_ receptor antagonist or genetic reduction of H_2_-histamine receptor levels would be a reasonable testable hypothesis for clinical studies. Moreover, drugs with histamine H_2_ agonistic properties are probably to be avoided in patients already suffering from arrhythmias. If the current data mimic the human situation, there is little concern that morphine, fentanyl, or ketamine can induce arrhythmia by releasing cardiac histamine. It is conceivable that patients using H_2_ antagonists for other indications (e.g., cimetidine in gastrointestinal diseases) might unintentionally benefit from their antiarrhythmic properties on H_2_-histamine receptors in the heart. Indeed, there have been sparse reports of antiarrhythmic effects of H_2_-histamine receptor antagonists in humans (Piotrowski et al. 2014).

## Limitations

We have not performed electrophysiological experiments like measuring action potentials or LTCC currents in right or left atrial or ventricular cardiomyocytes from H_2_-TG or WT to delineate underlying pathomechanisms in more detail. We also did not yet have the opportunity to perform telemetric studies in living H_2_-TG before and after injection of histamine, in order to find out whether we can induce arrhythmias in vivo in H_2_-TG compared to littermate controls. One can ask about the level of cardiac expression of H_2_ (and H_1_) receptors in the wild-type mouse heart, keeping in mind that in another rodent, namely the rat, H_2_ receptor levels are very low compared to levels in in the human heart (Matsuda et al. 2004). In brief, the mouse heart seems to be similar to the rat heart in this regard. When we prepared RNA from total heart tissue, we could detect with quantitative polymerase chain reactions H_1_ and H_2_ receptor mRNAs in the mouse heart (Gergs et al. 2019). However, we have not succeeded of H_2_ receptors in RNA prepared from mouse wild-type cardiomyocytes (unpublished observations). Hence, it is clear that the RNA for the H_2_ receptor is present in the heart of WT. However, it is unclear whether it is present in wild-type cardiomyocytes. We failed to find a commercial antibody that selectively detects the H_2_ receptor (Gergs et al. 2019). This is a well-known problem with G-protein-coupled receptors (Seifert et al. 2013). Moreover, using a radiolabeled H_2_ receptor agonist, we could detect the H_2_ receptor in H_2_-TG (Gergs et al. 2019) and also using immunohistology (Gergs et al. 2019). Hence, we are convinced that there is at least no H_2_ receptor coupled to force generation in any part of the wild-type adult mouse heart. But more work is needed in this regard.

At least, by this study, we can offer the scientific community a new model to test drugs to treat histamine-induced supraventricular and ventricular arrhythmias. Such follow-up studies might identify new drugs to treat human arrhythmias.

## Supplementary Information

Below is the link to the electronic supplementary material.

Supplementary file1 (PZF 910 KB)

Supplementary file2 (PZF 1230 KB)

Supplementary file3 (PZF 281 KB)

## Data Availability

Upon request.
